# Aquatic Birnavirus-Induced ER Stress-Mediated Death Signaling Contribute to Downregulation of Bcl-2 Family Proteins in Salmon Embryo Cells

**DOI:** 10.1371/journal.pone.0022935

**Published:** 2011-08-25

**Authors:** Hui Ling Huang, Jen Leih Wu, Mark Hung Chih Chen, Jiann Ruey Hong

**Affiliations:** 1 Laboratory of Molecular Virology and Biotechnology, Institute of Biotechnology, National Cheng Kung University, Tainan, Taiwan; 2 Laboratory of Marine Molecular Biology and Biotechnology, Institute of Cellular and Organismic Biology, Academia Sinica, Taipei, Taiwan; 3 Bioluminescence in Life-image Laboratory, Institute of Biotechnology, Department of Biotechnology, Hungkuang University, Taichung, Taiwan; University of Hong Kong, Hong Kong

## Abstract

Aquatic birnavirus induces mitochondria-mediated cell death, but whether connects to endoplasmic reticulum (ER) stress is still unknown. In this present, we characterized that IPNV infection triggers ER stress-mediated cell death via PKR/eIF2α phosphorylation signaling for regulating the Bcl-2 family protein expression in fish cells. The IPNV infection can induce ER stress as follows: (1) ER stress sensor ATF6 cleavaged; (2) ER stress marker GRP78 upregulation, and (3) PERK/eIF2αphosphorylation. Then, the IPNV-induced ER stress signals can induce the CHOP expression at early (6 h p.i.) and middle replication (12 h p.i.) stages. Moreover, IPNV-induced CHOP upregulation dramatically correlates to apparently downregulate the Bcl-2 family proteins, Bcl-2, Mcl-1 and Bcl-xL at middle replication stage (12 h p.i.) and produces mitochondria membrane potential (MMP) loss and cell death. Furthermore, with GRP78 synthesis inhibitor momitoxin (VT) and PKR inhibitor 2-aminopurine (2-AP) treatment for blocking GRP78 expression and eIF2α phosphorylation, PKR/PERK may involve in eIF2α phosphorylation/CHOP upregulation pathway that enhances the downstream regulators Bcl-2 family proteins expression and increased cell survival. Taken together, our results suggest that IPNV infection activates PKR/PERK/eIF2α ER stress signals for regulating downstream molecules CHOP upregulation and Bcl-2 family downregulation that led to induce mitochondria-mediated cell death in fish cells, which may provide new insight into RNA virus pathogenesis and disease.

## Introduction

The endoplasmic reticulum (ER) is a eukaryotic organelle that plays a vital role in a variety of cellular functions, including posttranslational modification, folding and synthesis of newly membrane and secretary proteins, metabolism, cellular calcium storage, and apoptosis [1]–[3]. Several stimuli, such as the accumulation of unfolded, misfolded, or excessive protein, oxidative stress, perturbation in calcium homeostasis, and virus infection can disrupt ER homeostasis and induce ER stress [4]–[7]. Protein aggregation is toxic to cells and consequently, numerous pathophysiological conditions are associated with R stress, including ischaemia, neurodegenerative diseases and diabetes [2]. In current knowledge, GRP78 is the master regulator of three UPR pathway via ER transmembrane receptors, including pancreatic ER kinase (PKR)-like ER kinase (PERK), activating transcription factor 6 (ATF6), and inositol-reguiring enzyme 1 (IRE1) [8]. However, if protein aggregation is persistent and the stress cannot be resolved, signaling switches from pro-survival to pro-apoptotic. The molecular mechanisms that facilitate this switch are now emerging [8].

Up to now, initiation phase of ER stress-induced apoptosis, dissociation of GRP78 from PERK initiates the dimerization and autophosphorylation of the kinase and generates active PERK. Once activated, PERK phorphorylates eukaryotic initiation factors 2α (eIF2), which results in attenuating global translation initiation and protein synthesis [9]. Then, in commitment phase of ER stress-induced apoptosis, CHOP, also as growth-arrest- and DNA-damage-inducible gene (GADD153), was originally identified in response to DNA damage. However, CHOP induction is probably most sensitive to ER stress condition [10]. Furthermore, to upregulate CHOP protein expression the PERK-eIF2α-ATF4 branch of the UPR is essential that promotes apoptosis and its suppressing by the activity of antiapoptotic Bcl-2 family proteins [10]. In the execution phase of ER stress-induced apoptosis, the cohort of caspases linked to ER stress-induced apoptosis has not yet been conclusively established, which triggers from death-receptor and mitochondria apoptotic pathways. Processing caspases-12, -3, -6, -7, -8 and -9 has been observed in different studies of ER stress, but caspase-12 has been proposed as a key mediator of ER stress-induced apoptosis [11].

Recently, some reports viruses [12]–[15] can induce ER stress, which also regulate viral replication or pathogenesis to decide cell survival or cell death [15]–[18]. Some examples of mouse retrovirus [13], hepatitis C virus [15] and Japanese encephalitis virus (JEV) [17] also can induce the ER stress response via upregulation of ER chaperon GRP78. Few of cases are focused on PERK/PKR pathway [12], [16], CHOP/GADD153 [14]. Further, a few cases of respiratory syncytial virus and simian virus 5 can induce apoptotic cell death through caspase-12 activation [18]–[20]. However, not much is known about ER stress responses to aquatic virus infection. The mechanism by which cells undergo apoptosis or are rescued from ER stress is also not well understood. Such studies could be useful in elucidating pathways involved in viral pathogenesis.

The Bcl-2 family of proteins, comprised of both anti- and pro-apoptotic molecules, constitutes a critical, intracellular decision point regulating a common death pathway [21]. The ratio of antagonist (Bcl-2, Bcl-x_L_, Mcl-1, and A1) to agonist (Bax, Bak, Bcl-x_s_ and Bad) molecules dictates whether a cell responds to a proximal apoptotic stimulus [21]. These proteins also interact with mitochondria to control the balance of mitochondrial membrane potential (MMP) [22], [23].

Infectious pancreatic necrosis virus (IPNV) is a fish pathogen and the prototype of the *Birnaviridae* virus family [24]. Birnaviruses possess a bi-segmented, double-stranded RNA genome contained within a medium-sized, unenveloped, icosahedral capsid. Gene expression involves the production of four unrelated major genes, which undergo various post-translational cleavage processes to generate three to five different structural proteins [25]. The largest of these proteins (VP1; 90–110 kDa) is encoded by the smaller segment B RNA [26]. The larger genome segment A: A large open reading frame (ORF) encodes VP3 (submajor capsid protein; 32 kDa) that play a new role as a death factor [27], VP4 (28 kDa), and VP2 (major capsid protein; 46 kDa) [28]; and small ORF encodes a small non-structural protein VP5 (17 kDa), which play an anti-apoptotic function [28], [29].

Previously, IPNV infection may induce apoptosis in a fish cell line [30]–[33], cell death may be through activation of caspase-8 and -3 [34], and apoptosis requires new protein synthesis [32], which acts through NF-κB transcription factor activation for trans-activating the downstream effector genes such as Bad [35]. Recently, IPNV infection can induce mitochondrial membrane potential (MMP) loss, which block by ANT inhibitor BKA [36] and IPNV-induced expression gene annexin 1 could play anti-death function [37]. On the other hand, IPNV can increase eIF2α phosphorylation is via interferon/eIF2α/PKR response in RTG-2 cells [38], but they are how to trigger ER stress and how to regulate the mitochondria function is remain unknown.

In this present, we examined that IPNV infection triggers PKR/PERK/eIF2α ER stress response that can involve in CHOP upregulation and Bcl-2 family members downregulation, which connect to regulate the mitochondria function. From our finding, that may provide new insights into RNA virus pathogenesis via ER stress response regulates host cell death.

## Materials and Methods

### Cell line and virus

Chinook salmon embryo cells (CHSE-214) were obtained from the American Type Culture Collection (ATCC, Manassas, VA, USA). Cells were grown at 18°C in plastic tissue-culture flasks (Nalge Nunc International, Rochester, NY, USA) containing Eagle's minimum essential medium (MEM) supplemented with 10% (v/v) fetal bovine serum (FBS) and gentamicin (25 µg/ml). An isolate of the Ab strain of IPNV, designated E1-S, was obtained from Japanese eels in Taiwan [39]. The virus was propagated in CHSE-214 cell monolayers at a multiplicity of infection (MOI) of 0.01 per cell. Infected cultures were monitored as described previously [40] and TCID_50_ assay was performed on confluent monolayers [41].

### Western-blot analysis

Monolayers of CHSE-214 cells (4.0 ml, 10^5^ cells per ml) on 60-mm Petri dishes were cultivated for at least 20 h and rinsed twice with PBS. In viral protein expression assay, cells were infected with virus (MOI = 1) and incubated for 0 h, 6 h or 12 h p.i.; In GRP78 inhibitor vomitoxin (VT, 1 µg/ml; Sigma Chemicals, MO, USA) assay, pretreated two hours then incubated for 0 h, 6 h, or 12 h p.i. that vomitoxin have some side effects on causing a ribotoxic stress response, inhibiting protein synthesis and in some systems activating PKR; In PKR inhibitor 2-aminopurine (2-AP; 20 mM; Sigma Chemicals, MO, USA) assay, pretreated two hours then incubated for 12 h p.i. that 2-aminopurine may function in general as an ATP analogue and has effects art multiple levels and on may kinases. At the end of each incubation period, the culture medium was aspirated, and the cells were washed with PBS and then lysed in 0.3 ml of lysis buffer (10 mM Tris base, 20% glycerol, 10 mM sodium dodecyl sulfate, and 2% β-mercaptoethanol; pH = 6.8).

Proteins were separated by SDS-polyacrylamide gel electrophoresis [42], electroblotted, and subjected to immunodetection as described elsewhere [43]. Blots were incubated with a 1∶3000 dilution of anti-IPNV E1-S particle polyclonal antibodies (provided by Dr. Wu), and a 1∶10000 dilution of a peroxidase-labelled goat anti-rabbit conjugate (Amersham, Piscataway, NJ, USA); or with a 1∶2000 dilution of anti-mouse GRP78 (BD Biosciences, Palo Alto, CA 94303-4230, USA), PERK (ROCKLAND), PERK phosphorylation (BioLegend, San Diego, California, USA), eIF2α(Cell Signaling Technology, Danvers, MA 01923, USA), eIF2α phosphorylation (Cell Signaling Technology), CHOP (BioLegend), Bcl-2 (BD Biosciences), Mcl-1 (CHEMICO), Bcl-xL (BD Biosciences) and actin monoclonal antibodies (BD Biosciences), and a 1∶8000 dilution of a peroxidase-labelled rabbit anti-mouse conjugate.

Chemiluminescence detection was performed according to the instructions provided with the Western Exposure Chemiluminescence Kit (Amersham). The chemiluminescence was visualized by exposure to Kodak XAR-5 film (Eastman Kodak, Rochester, NY, USA).

### PS exposure assay

Monolayers of CHSE-214 cells (4.0 ml, 10^5^ cells per ml) on 60-mm Petri dishes were cultivated for at least 20 h and rinsed twice with PBS. Cells were infected with virus (MOI = 1) and incubated for 0 and 12 h p.i. On the other hand, the cells pretreated GRP78 inhibitor vomitoxin (VY, 1 µg/ml) for 2 h, then cells were infected with virus (MOI = 1) and incubated for 0, 6 h and 12 h p.i. In early apoptotic cell assay (Annexin V-FLUOS staining or Annexin V-Red; 29): Exposure of PS on the outer leaflet of early apoptotic cell membranes was analyzed using annexin V-fluorescein to differentiate apoptotic from non-apoptotic cells. At 0, 8 and 12 h post-infection time (h p.i.), cells were removed from the medium, washed with PBS, and then incubated with 100 µl of a commercially available staining solution (annexin V-fluorescein in a HEPES buffer; Boehringer-Mannheim, Mannheim, Germany) for 10–15 min. Evaluation was by fluorescence microscopy (Olympus IX 70; Halagaya Shibuta-ku, Tokyo, Japan) using a 488-nm excitation wavelength and 515-nm long-pass filter for detection [30]. Each group sample (two dishes) was counted three times, and each time, 200 or more cells were counted. The characteristics of cells were recorded according to the colour and structure of the cell. The mean of the three counts of each different cell characteristic was used to calculate the apoptotic and necrotic cell indices and their respective bars.

### Evaluation of mitochondrial membrane potential with a lipophilic cationic dye

CHSE-214 cells (4.0 ml, 10^5^/ml in a 60-mm Petri dish) were cultured as monolayers for 20 h and then rinsed twice with phosphate-buffered saline (PBS). To evaluate IPNV induces mitochondria-mediated cell death. CHSE-214 cells were infected with E1-S strain and then incubated for 0, 6 h, and 12 h p.i. By contrast, to evaluate VT treatment, cells were pretreated GRP78 inhibitor vomitoxin (VY, 1 µg/ml) for 2 h and then incubated for 0, 8 h, 12 h and 24 h p.i.; and in Bcl-xL overexpression stably cells and EGFP cells were infected with virus (MOI = 1) and incubated for 0, 12 h and 24 h p.i. For assessment of mitochondrial membrane potential (ΔΨm), CHSE-214, EGFP-Bcl-xL-2, EGFP-3, VT plus CHSE-214 cells were stained using MitoCapture reagent (500 µl per dish incubated at 37°C for 15–20 min; BioVision, Mountain View, CA, USA). This lipophilic cationic dye accumulates and aggregates in mitochondria when ΔΨm is normal and remains in the cytoplasm when it is not. Loss of fluorescence intensity observed under fluorescence microscopy was taken as a marker of mitochondrial membrane disruption and reduced potential [44]. Evaluation was by fluorescence microscopy using a 488-nm excitation wavelength and 515-nm long-pass filter for detection of fluorescein, and using a 510-nm excitation wavelength and 590-nm long-pass filter for detection of rhodamine.

### Caspases activity assay (caspase-3)

#### Intact cell assay

About 10^5^ CHSE-214 cells/ml were seeded in a 60-mm Petri dish from Nunc (Nalge Nunc International, Rochester, NY, USA) and cultured for 20 h at 18°C. The cells pre-treated VT for 2 h, then infected with IPNV and in cells for caspase-3 (PhiphiLux-G_2_D_2,_ red; OncoImmunin, TM, USA) assays, then further incubation for 0, and 12 h p.i. At the end of each time point, substrate was present at 10 µM for one hour at 18°C, then evaluation was by fluorescence microscopy using a 488-nm excitation wavelength and 515-nm long-pass filter for detection of fluorescein, and using a 510-nm excitation wavelength and 590-nm long-pass filter for detection of rhodamine. Each group sample (two wells) was counted three times, and each time, 200 or more cells were counted. The characteristics of cells were recorded according to the colour and structure of the cells. The mean of the three counts of each different cell characteristic was used to calculate the caspase-3 activation cell indices and their respective bars.

### Preparation of mitochondria from CHSE-214 cells

CHSE-214 cells (10 ml, 10^5^/ml in a 100-mm Petri dish) were cultured as monolayers for 20 h and then rinsed twice with phosphate-buffered saline (PBS). To evaluate IPNV induces mitochondria-mediated cell death. CHSE-214 cells were infected with E1-S strain and then incubated for 12 h p.i. By contrast, to evaluate VT treatment, cells were pretreated GRP78 inhibitor vomitoxin (VT, 1 µg/ml) for 2 h and then were infected with virus (MOI = 1) and incubated for 0, 12 h p.i. At each time point subsequent to a change of the culture medium, 1 ml was removed. Mitochondria were isolated by a modification of a previously described protocol [44]. Briefly, CHSE-214 cells (2×10^6^) were washed with PBS and homogenized in 0.3-ml of mitochondria isolation buffer (0.35 M mannitol, 10 mM HEPES, 0.1% bovine serum albumin, pH 7.2) using a glass homogenizer. Unbroken cells and nuclei were pelleted by centrifugation (600× *g* for 5 min at 4°C). The mitochondria pellet was isolated from centrifuged supernatant (10,000× *g* for 10 min at 4°C) and supernatant was collected and mixed with 25 µl of 10× sodium dodecyl sulfate sample buffer. Samples (50 µl) were boiled and subjected to Western blot analysis [42]–[43].

### Nuclear protein extraction from CHSE-214 cells

CHSE-214 cells (4.0 ml, 10^5^/ml in a 60-mm Petri dish) were cultured as monolayers for 20 h and then rinsed twice with phosphate-buffered saline (PBS). To evaluate IPNV induces CHOP expression in CHSE-214 cells. CHSE-214 cells were infected with E1-S strain and then incubated for 12 h p.i. By contrast, to evaluate VT treatment, cells were pretreated GRP78 inhibitor vomitoxin (VY, 1 µg/ml) for 2 h and then were infected with virus (MOI = 1) and incubated for 0, 12 h p.i. At each time point subsequent to a change of the culture medium, 1 ml was removed. Mitochondria were isolated by a modification of a previously described protocol [44].

### Cell counts

Loss of MMP and percentage of annexin V-fluorescein positive cells were determined in each sample by counting 200 cells. Each result was expressed as mean ± SEM. Data were analyzed using either paired or unpaired Students *t* tests, as appropriate. A value of *p*<0.05 was taken to represent a statistically significant difference between group mean values.

## Results

### IPNV infection can activate ER stress sensor ATF-6 and up-regulation of chaperone GRP78 at early replication (6 h p.i.) stage in CHSE-214 cells

**Figure 1:**
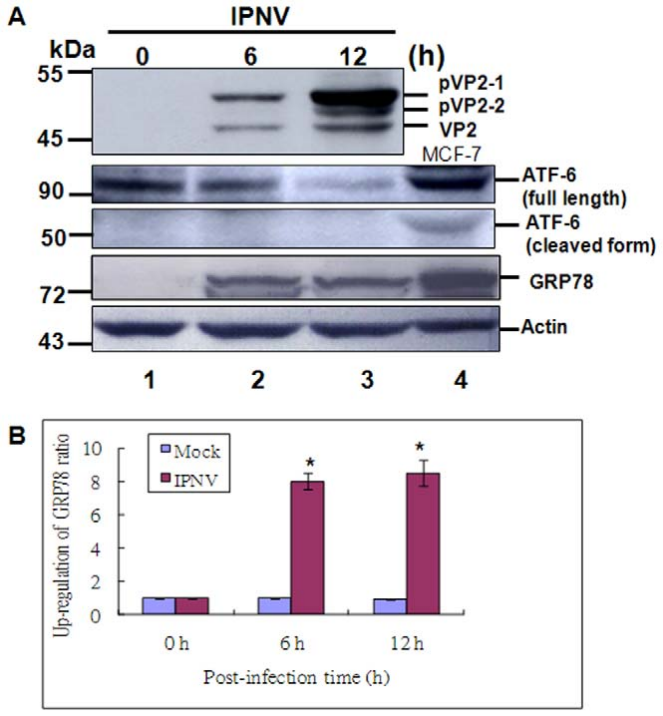
Identification of IPNV infection induces ER stress sensor ATF6 cleavage and up-regulate marker GRP78 in CHSE-214 cells. Infection of IPNV E1S (MOI = 1) in CHSE-214 cells following incubation for 0 h, 6 h, and 12 h p.i. (A) VP2, ATF6 and GRP78 proteins were detected by Western blots, and the gels were immunoblotted with a polyclonal antibody to whole particle IPNV E1-S whole particle, with a monoclonal antibody to anti-mouse ATF6 and GRP78. Lanes 1–3: Cells infected with IPNV were incubated for 0 h (lane 1), 6 h (lane 2), and 12 h (lane 3). The MCF-7 cell lysate is a positive control as shown in lane 4. Internal control Actin for experiments whose results are depicted in panel A. (B) Quantification of ER stress marker GRP78 upregulation levels in IPNV-infected cells at early and middle replication stages. Protein expression level was quantified by Personal Densitometer (Molecular Dynamic). Data were analyzed using either paired or unpaired Student's t-tests, as appropriate. * *p*<0.05 was taken as a statistically significant difference between group mean values.

Recently, some reports viruses [12]–[15] can induce ER stress, which also regulate viral replication or pathogenesis to decide cell survival or cell death [15]–[18], but RNA virus induced-ER stress signaling directly effect mitochondria function is remain uncover. We try to ask IPNV infection whether can induce ER stress. In figure 1 shows the expression profile of the major capsid protein VP2, which was gradually processed from its precursor form pVP2-1 (52 kDa) and intermediate form pVP2-2 (50 kDa) to mature VP2 (46 kDa) at 6 h to 12 h p.i. (Fig. 1A, lanes 2–3) in infected CHSE-214 cells as compare with uninfected cells; Fig. 1A, lane 1 at 0 h as a negative control. Furthermore, at 6 h and 12 h p.i, IPNV infection can either gradually induce ER stress sensor ATF-6 cleavage (Fig. 1A, lanes 2–3) or can upregulate GRP78 (Fig. 1A, lanes 2–3) as compared with 0 h (Fig. 1A, lane 1) as a control group (Fig. 1A, lane 1). The positive control MCF-7 cell lysate is shown in Fig. 1, lane 4. The internal control β-actin is shown in Fig. 1A, lanes 1–4. The upregulation ratio of GRP78 are 8 folds (6 h p.i.) and 8.5 folds (12 h p.i.) as shown in Fig. 1B as compared with 0 h that based level is as a one-fold. These results suggest that IPNV infection can trigger UPR at 6 h p.i. (early replication stage) in this fish system.

### IPNV infection can activate PERK/eIF2 α phosphorylation ER signal and upregulation of CHOP

Furthermore, IPNV triggered ER stress whether can activate PERK is still unknown.

**Figure 2:**
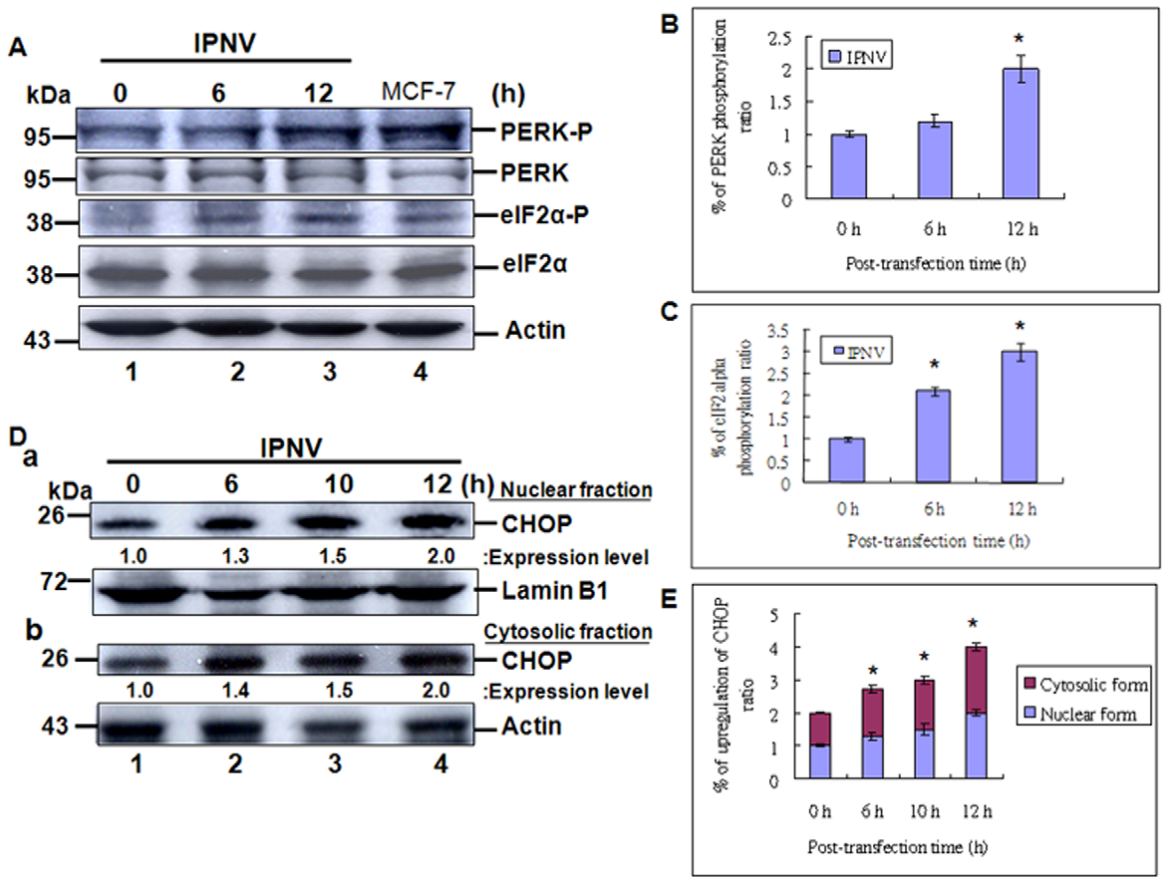
Identification of IPNV-induced ER stress signal can phosphorylate the PERK and eIF-2α protein and upregulate CHOP protein in CHSE-214 cells. Infection of IPNV in CHSE-214 cells following incubation for 0 h, 6 h, 10 h and 12 h p.i. (A) PERK and eIF2α proteins and its phosphorylated levels were detected by Western blots, and the gels were immunoblotted with a polyclonal antibody to mouse phosphor-PERK (Thr980), PERK, phosphor-eIF2α (Ser51) and eIF2α. Lanes 1–3: Cells infected with IPNV were incubated for 0 h (lane 1), 6 h (lane 2), and 12 h (lane 3). The MCF-7 cell lysate is a positive control as shown in lane 4. Internal control Actin for experiments whose results are depicted in panel A. (B–C) Quantification of phosphor-PERK (Thr980) and phosphor-eIF2α (Ser51) phosphorylation upregulation levels in IPNV-infected cells at early and middle replication stages. Protein expression level was quantified by Personal Densitometer (Molecular Dynamic). Data were analyzed using either paired or unpaired Student's t-tests, as appropriate. * *p*<0.05 was taken as a statistically significant difference between group mean values. (D) CHOP (GADD153), Lamin B1 and Actin expression levels were detected by Western blots, and the gels were immunoblotted with a polyclonal antibody to CHOP (GADD153) and Lamin B1, with a monoclonal antibody to Actin. D:a, lanes 1–4: Cells infected with IPNV were incubated in nuclear fraction samples for 0 h (lane 1), 6 h (lane 2), 10 h (3) and 12 h (lane 4). Nuclear control Lamin B1 for experiments whose results are depicted in panel D:a. D:b, lanes 1–4: Cells infected with IPNV were incubated in cytosolic fraction samples for 0 h (lane 1), 6 h (lane 2), 10 h (3) and 12 h (lane 4). Cytosolic control Actin for experiments whose results are depicted in panel D:b. (E) Quantification of CHOP expression levels in IPNV-infected cells at early and middle replication stages. Data were analyzed using either paired or unpaired Student's t-tests, as appropriate. * *p*<0.05 was taken as a statistically significant difference between group mean values.

In our system, we found that IPNV infection just can mild activate the PERK at 6 h (Fig. 2A:a, lane 2; 2A:b) and apparently activated at 12 h (Fig. 2A:a, lane 3) p.i., as compared with mock control (Fig. 2A, lane 1, at 0 h) and positive control (Fig. 2A:a, lane 4). The activation of PERK further apparently phosphorylate its substrate eIF2α at 6 h (Fig. 2A:a, lane 2) and 12 h (Fig. 2A:a, lane 3) p.i. as compared with mock control (Fig. 2A:a, lane 1, at 0 h) and positive control (Fig. 2A:a, lane 4). The un-phosphorylation of eIF-2 and actin as a internal control are shown in Fig. 2A:a, lanes 1–3. The PERK autophosphorylation ratios are shown in Fig. 2B that are gradually increased from one fold (0 h) to 1.2 folds (6 h p.i.) and 2.0 folds (12 h p.i.). The eIF2α phosphorylation ratio by PERK are quickly increased from one fold (0 h) to 2.1 folds (6 h p.i.) and 3.0 folds (12 h p.i.). Furthermore, In nuclear fraction (Fig. 2D:a) and cytosoloic fraction (Fig. 2D:b) also found that PERK/eIF2α signaling can upregulate the CHOP protein expression at 6 h p.i. (2.7 folds), 10 h p.i. (3.0 folds) and 12 h p.i. (4.0 folds) in total amount of expression level that includes nuclear and cytosolic fractions (Fig. 2E) as compared with 0 h (1 fold).

These data suggest that eIF2 phosphorylation can regulate the downstream CHOP expression at early and middle replication stages.

### IPNV-induced eIF2α/CHOP death signaling can affect Bcl-2 family protein expressions

**Figure 3:**
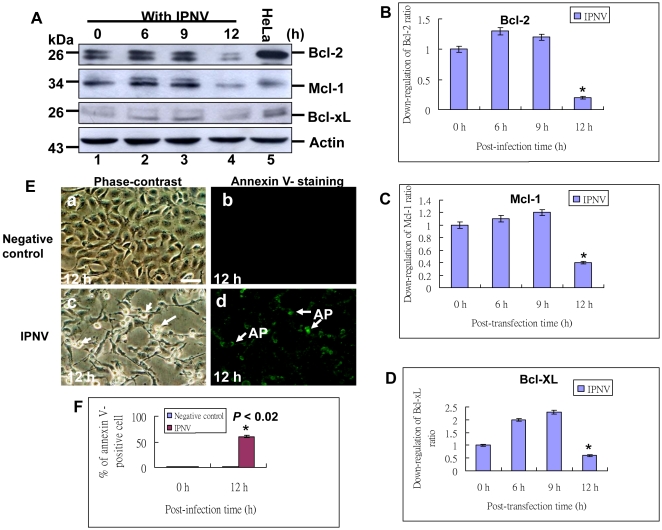
Identification of IPNV-induced ER stress signal can induce Bcl-2 family proteins downregulation and apoptotic cell death. Infection of IPNV in CHSE-214 cells following incubation for 0 h, 6 h, 9 h and 12 h p.i. (A) Bcl-2, Mcl-1 and Bcl-xL proteins expression levels were detected by Western blots, and the gels were immunoblotted with a polyclonal antibody to Bcl-2 and Bcl-xL, and with a monoclonal antibody to Mcl-1. Lanes 1–4: Cells infected with IPNV were incubated for 0 h (lane 1), 6 h (lane 2), 9 h (lane 3) and 12 h (lane 4). The HeLa cell lysate is a positive control as shown in lane 5. Internal control Actin for experiments whose results are depicted in panel A. (B–D) Quantification of Bcl-2, Mcl-1 and Bcl-xL proteins expression levels in IPNV-infected cells at early and middle replication stages. Protein expression level was quantified by Personal Densitometer (Molecular Dynamic). Data were analyzed using either paired or unpaired Student's t-tests, as appropriate. * *p*<0.05 was taken as a statistically significant difference between group mean values. (E) Identification of IPNV induces apoptotic and post-apoptotic necrosis in CHSE-214 cells. Annexin V-labeled (fluorescing) apoptotic cells indicated by arrows at 12 h p.i. with IPNV infection. (Fig. 3E, panels c and d); without infection group as negative control (Fig. 3E, panels a and b) at 12 h p.i. (Bar = 10 µm.). (F) Annexin-V positive cells were counted per 200 cells and data was analyzed using either or unpaired Student *t*-tests as appropriate at 0 and 12 h p.i. A value of *p*<0.02 was taken to represent a statistically significant difference between mean values of groups.

Furthermore, as we known to upregulate CHOP protein expression the PERK-eIF2α-ATF4 branch of the UPR is essential that promotes apoptosis. In our system, dramatically found that CHOP upregulation can connect to suppress the Bcl-2 family proteins as Bcl-2, Mcl-1 and Bcl-xL, which also suppress the host cell apoptotic cell death (21–23). In western blot analysis, the Bcl-2 families were also mild upregulated at 6 h p.i. (Fig. 3A, lane 2) and 9 h p.i. (Fig. 3A, lane 3) as compared with negative control (0 h; Fig. 3A, lane 1) and positive control HeLa cell lysate (Fig. 3A, lane 5), but then dramatically downregulated their proteins expression at 12 h (Fig. 3A, lane 4) p.i. that up- and downregulation ratios were shown in Fig. 3B–D for tracing Bcl-2, Mcl-1 and Bcl-xL expression profiles, respectively. The Bcl-2 family proteins downregulation was correlated to host cell death (Fig. 3E: c–d; indicated by arrows) as compared with negative control (Fig. 3E: a–b) by Annexin V-staining at 12 h p.i. that Annexin V-positive cell ratio was shown in Fig. 3F, which was up to 50% than negative control. These data suggest that IPNV infection-triggered eIF2α/CHOP signaling significantly involved in downregulation of Bcl-2 family proteins as Bcl-2, Mcl-1 and Bcl-xL and cell death.

### Dual signal of PERK/PKR regulates eIF2α phosphorylation with IPNV infection

**Figure 4:**
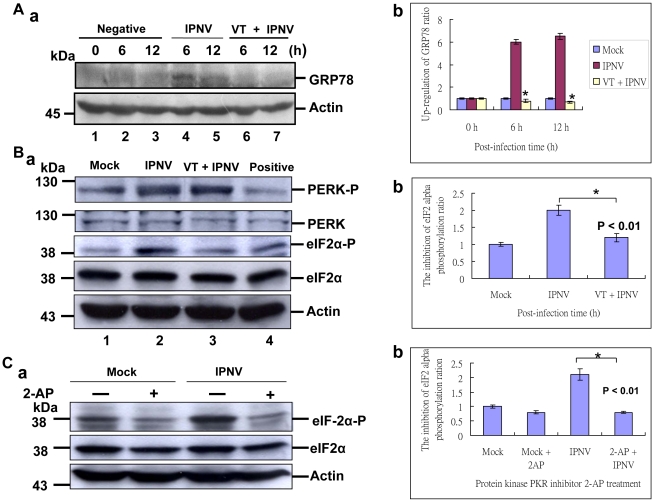
Identification of PKR/PERK two signal pathways on eIF2 phosphorylation in CHSE-214 cells with IPNV infection. Pretreatment chaperone GRP78 inhibitor VT (1 µg/mL) with CHSE-214cells for two hours, then infection of IPNV in CHSE-214 cells following incubation for 0 h, 6 h and 12 h p.i. (A:a) western blot analysis of GRP78 protein expression in IPNV-infected CHSE-214 cells. IPNV-infected CHSE-214 cells at 6 h p.i. (lane 4) and at 12 h p.i. (lane 5); with VT (1 µg/mL) treatment at 6 h p.i. (lane 6), and at 12 h p.i. (lane 7); uninfected cells at 0 h p.i. (lane 1), 6 h (lane 2) and 12 h (lane 3). Actin was used as an internal control in lanes 1–7. Quantification of GRP78 expression levels in IPNV-infected cells at early and middle replication stages. (A:b) Protein expression level was quantified by Personal Densitometer (Molecular Dynamic). Data (three times independent experiments) was analyzed using either or unpaired Student *t*-tests as appropriate at 0, 6 and 12 h pi. A value of *p*<0.05 was taken to represent a statistically significant difference between mean values of groups. (B:a) western blot analysis of PERK and eIF2α protein phosphorylation level in IPNV-infected CHSE-214 cells at 12 h p.i.; lane 1 without IPNV-infected cells; lane 2, IPNV-infected CHSE-214 cells; lane 3, with VT (1 µg/mL) treatment and IPNV-infected CHSE-214 cells; lane 4, with ER stress drug A23187 treatment for 2 hours. Actin was used as an internal control in lanes 1–4. (B:b) Quantification of eIF2α protein phosphorylation level in IPNV-infected cells at middle replication stage. Protein expression level was quantified by Personal Densitometer (Molecular Dynamic). (C:a) western blot analysis of eIF2α protein phosphorylation level in IPNV-infected CHSE-214 cells at 12 h pi. IPNV-without infected cells (lane 1); with protein kinase inhibitor 2-aminopurine (2-AP; 20 mM) pretreatment two hours CHSE-214 cells; with IPNV-infected CHSE-214 cells (lane 3); with 2-AP treatment plus IPNV-infected CHSE-214 cells (lane 4). Actin was used as an internal control in lanes 1–4. (C:b) Quantification of eIF2α protein phosphorylation level in IPNV-infected cells at middle replication stage. Protein expression level was quantified by Personal Densitometer (Molecular Dynamic).

In our system, IPNV infection may upregulate the chaperone protein GRP78 (Fig. 1A) for modulate the unfold proteins during a large amount of viral protein expressions. Then, we try to block GRP78 induction whether connects to reduce ER stress. The results of GRP78 inhibitor vomintoxin (VT; 1 µg/ml) treatment, can effectively block the GRP78 upregulation at 6 h (Fig. 4A:a, lane 6) and 12 h (Fig. 4A:a, lane 7) p.i. as compare with without treatment group at 6 h (Fig. 4A:a, lane 4) and 12 h (Fig. 4A:a, lane 5) p.i. and negative control at 0 h, 6 h and 12 h (Fig. 4A:a, lanes 1–3), which also apparently blocked GRP78 expression up to 6 folds as shown in Fig. 4A:b at 6 h and 12 h p.i. Then, we found that VT can block GRP78 induction but did not inhibit the PERK phosphorylation (Fig. 4B:a, lane 3) at 12 h pi when compared with IPNV infection group (Fig. 4B:a, lane 2) and negative control (Fig. 4B:a, lane 1). On the other hand, it is very interesting that VT did block PERK phosphorylation, but can inhibit eIF2α phosphorylation (Fig. 4B:a, lane 3) from two folds down to one fold (Fig. 4B:b) as compared with mock group and VT plus IPNV group. So, we try to ask other possibility whether eIF2α phosphorylation is via PKR pathway. Then, we try to treat PKR specific inhibitor 2-AP for blocking eIF2α phosphorylation. The results of 2-AP (0.1 uM) can block eIF2α phosphorylation at 12 h p.i. (Fig. 4C:a, lane 4) when compared with without treatment group (Fig. 4C:a, lane 3) and negative control group (Fig. 4C:a, lanes, 1–2), which reduces phosphorylation ratio is apparently from 2.1 folds down to 0.8 fold (Fig. 4C:b). These data suggest that IPNV-induced ER stress signal for eIF2α phosphorylation, which phosphorylated by using dual signal (PERK/PKR) pathways during in the early and middle replication stages.

### IPNV infection-induced ER stress signal can upregulate CHOP and can downregulate Bcl-2 family proteins

**Figure 5:**
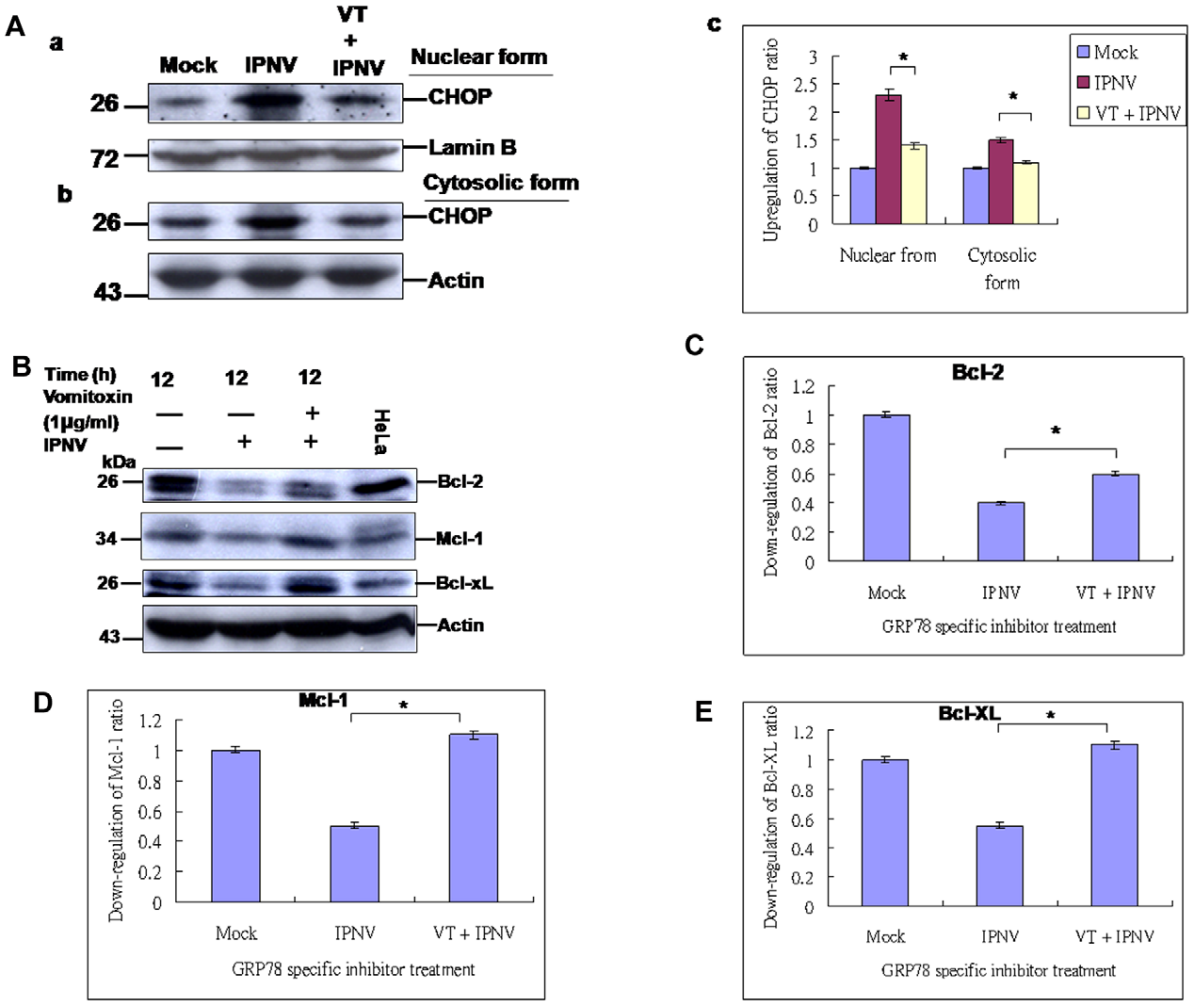
Identification of IPNV-induced ER stress signal can up-regulate CHOP and can downregulate the Bcl-2 family protein at middle replication stage. (A:a, nuclear form and b, cytosolic form) Western blot analysis of CHOP protein expression in without IPNV-infected CHSE-214 cells (lane 1), with IPNV-infected CHSE-214 cells (lane 2), with VT pretreatment and IPNV-infected CHSE-214 cells (lane 3) at 12 h p.i. Actin was used as an internal control in lanes 1–3. (A:c) Quantification of CHOP expression level in RGNNV-infected cells at middle replication stage. (B) Western blot analysis of Bcl-2. Mcl-1 and Bcl-xL protein expressions in IPNV-infected CHSE-214 cells with VT (1 µg/mL) treatment at 12 h p.i (lane 3); IPNV-infected CHSE-214 cells at 12 h pi (lane 2); uninfected cells at 12 h (lane 1); HeLa cell lysate as a positive control (lane 4). Actin was used as an internal control in lanes 1–4. (C–E) Quantification of Bcl-2, Mcl-1 and Bcl-xL expression levels in IPNV-infected cells at middle replication stage. Protein expression level was quantified by Personal Densitometer (Molecular Dynamic).

When unfold proteins continue to accumulate beyond the capacity of the ER, apoptosis may occur. Under the ER stress, CHOP is activated to facilitate cell death, which the downstream targets of CHOP remain unknown, but CHOP-mediated apoptosis has been coupled to a pathway that suppresses Bcl-2 expression, depletion of intracellular glutathionine, and an increase of free radicals [45]. In our system, IPNV-induced the PERK-eIF2α signaling pathway can upregulate the CHOP (Fig. 2D), but whether regulate the Bcl-2 family is still unknown. When treatment of VT for blocking GRP78, can correlate to dephosphorylate the eIF2α and reduces the CHOP expression (Fig. 5A:a and b, lane 3) at 12 h p.i. in nuclear fraction and cytosolic form as compared with IPNV infection group (Fig. 5A:a and b, lane 2) and negative control (Fig. 5A:a and b, lane 1), which downregulation ratio from 2.3 folds was down to 1.4 folds in nuclear form and from 1.5 folds was down to 1.1 folds as compared with mock control one fold each in nuclear and cytosolic form (Fig. 5A:c). Furthermore, downregulation of CHOP are increased the Bcl2 family protein expression levels such as Bcl-2, Mcl-1 and Bcl-xL (Fig. 5B, lane 3) as compared with IPNV infection group (Fig. 5B, lane 2) and mock group (Fig. 5B, lane 1) at 12 h p.i., which expression level of Bcl-2 increased from 0.4 folds to 0.6 folds (Fig. 5C); Mcl-1 increased from 0.5 folds to 1.1 folds (Fig. 5D); and Bcl-xL increased from 0.5 folds to 1.1 folds (Fig. 5E). The mock groups were as one fold (Fig. 5B, lane 1) for Fig. 5C–E.

### IPNV-induced ER stress signals affect the MMP loss, caspase-3 activation

**Figure 6:**
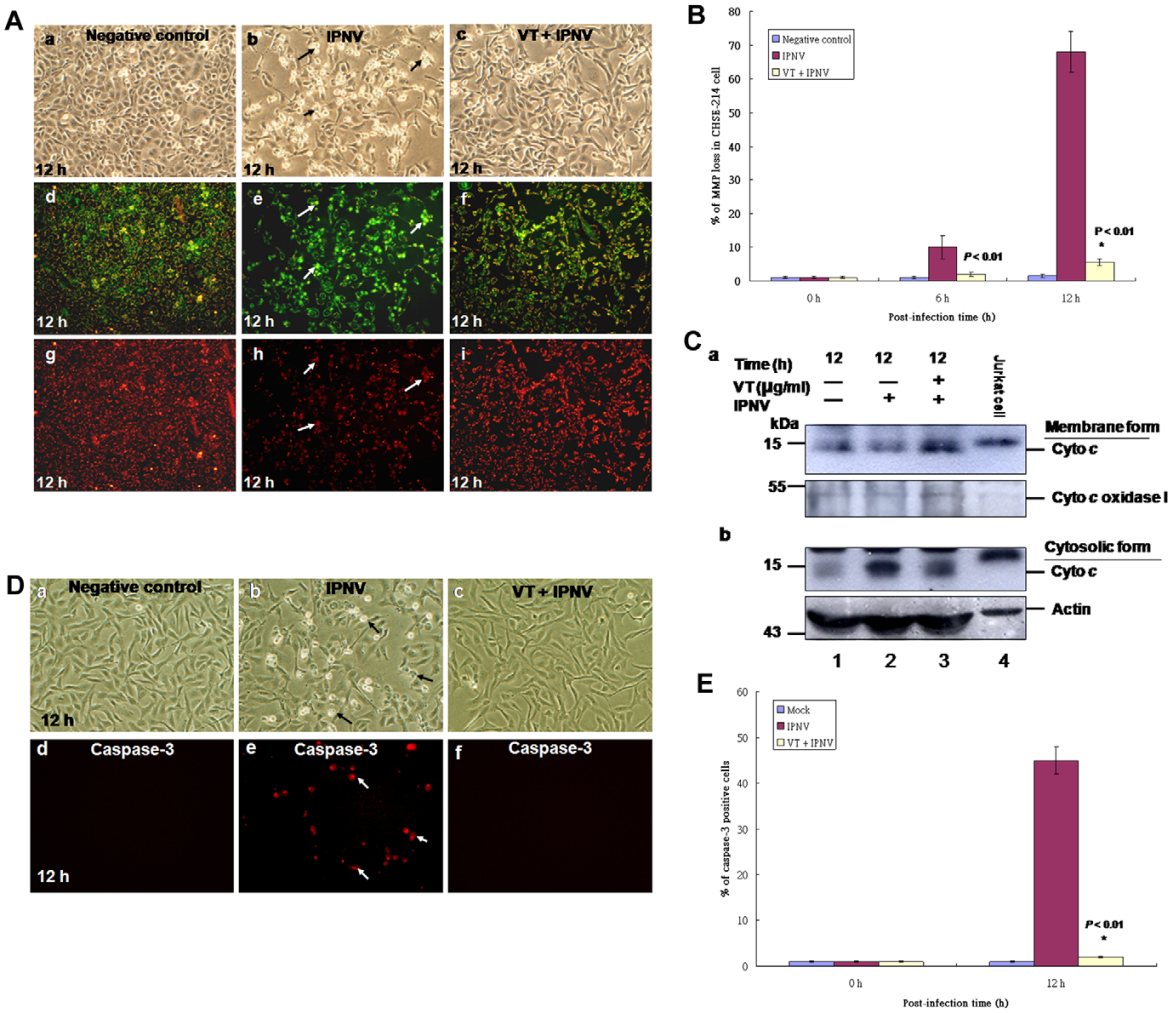
Inhibition of ER stress signal can reduce IPNV-induced MMP loss and caspase-3 activation. (A) MMP loss was demonstrated by either strong green fluorescence or loss of red fluorescence via inhibition of GRP78 expression by its inhibitor VT. Phase-contrast and fluorescence images of uninfected cells as a negative control (a, d and g) at 12 h; IPNV-infected cells (b, e and h) at 12 h; IPNV-infected with VT treatment cells (c, f and i) at 12 h that were stained with the lipophilic cationic dye at 12 h p.i.; loss of red fluorescent cells or strong green fluorescent cells are indicated by arrows. (B) The percentage loss of MMP was calculated at 0 h, 6 h and 12 h p.i. for uninfected, IPNV-infected cells and IPNV-infected with VT treatment cells. Data were analyzed using either paired or unpaired Student's t-tests, as appropriate. * *p*<0.05 was taken as a statistically significant difference between group mean values. (C) The release of cytochrome *c* in CHSE-214 cells is shown at 12 h pi. with uninfected (lane 1), IPNV-infected cells (lane 2), IPNV-infected with VT treatment cells (lane 3) and Jurkat cell lysate (lane 4). (Fig. 6C: a) the release of cytochrome *c* from mitochondrial membranes was detected in gels using Western immunoblotting with polyclonal antibodies against mouse cytochrome *c*. Internal control, cytochrome *c* oxidase I, is in Fig. 6C: a. (Fig. 6C:b) the cytosolic form cytochrome c was detected in gels using Western immunoblotting with polyclonal antibodies against mouse cytochrome c . Internal control, Actin, is in Fig. 6C: b. (D) Phase-contrast and fluorescent images of VT treatment blockade of IPNV induces effector caspase-3 activation in CHSE-214 cells by *in situ* assay. Treatment with VT blocked effector caspase-3 activation at 12 h p.i. Time course of cells incubated with 10 uM DEVDase ((PhiPhiLux-G_2_D_2_); red). Fluorescence images of caspase-3 in apoptotic CHSE-214 cell derived from DEVDase are shown separately to assess their differential activity, which caspase-3 positive cells in red color were indicated by arrows. (E) Fluorogenic substrate assays were performed in triplicate. Error bars represent standard error of the mean. Fluorescence is expressed as arbitrary units. Data were analyzed using either or unpaired Student *t*-tests as appropriate. A value of *p*<0.01 was taken to represent a statistically significant difference between mean values of groups.

Then, to modulate the eIF-2/CHOP signalings by VT for inhibition of GRP78 synthesis whether correlates to disrupt the mitochondria function is still unknown. To determine whether MMP loss was affected by IPNV infection in CHSE-214 cells, a mitochondrial function dye (JC-1 dye) was used to determine change in the MMP of IPNV-infected cells. The dye aggregates in the mitochondria of healthy cells and fluoresces red. In apoptotic cells, the dye cannot accumulate in mitochondria, remains as monomers in the cytoplasm, and fluoresces green. The VT treatment group can block IPNV-induced MMP loss in CHSE-214 cells (Fig. 6A: c, f and i) as compared with IPNV infection group (Fig. 6A: b, e and h; indicated by arrows) and negative control (Fig. 6A: a, d and g). The MMP loss ratio is shown in Fig. 6B, at 6 h p.i. 10% was down to 3%; 12 h p.i. 69% was down to 4% in VT plus IPNV group as compared with IPNV infection group. The negative control group is 1.5% and 2% at 6 h and 12 h p.i., respectively. Furthermore, we have found that the cytochrome *c* release in IPNV-infected cells from mitochondrial membrane form (Fig. 6C:a, lane 2) and cytosolic form (Fig. 6C: b, lane 2) at 12 h p.i. as compared with VT plus IPNV infection group in membrane form (6C:a, lane 3) and cytosolic form (Fig. 6C: b, lane 3) and negative control group membrane form (6C:a, lane 1) and cytosolic form (6C:a, lane 1). The Jurkat cell lysate as a positive control is shown in (6C:a and b, lane 4). The internal control cyto *c* oxidase I for membrane form is shown in Fig. 6C:a, lanes 1–3; Actin for cytosolic form is shown in Fig. 6C:b, lanes 1–3. In addition to determine whether caspase-3 activation was blocked by VY treatment. In caspase-3 activation assay, we found that VT treatment can effectively reduce caspase-3 activation (Fig. 6D: c and f) as compared with IPNV infection group (Fig. 6D: b and e; positive cell indicated by arrows) and negative control (Fig. 6D: a and d), which percentage of caspase-3 positive cell is shown in Fig. 6E. At 12 h p.i., 45% (IPNV infection group) was down to 2% (VT+IPNV) and negative control 1%. These data suggest that blockade of GRP78 upregulation by VT can reduce ER stress signal induces mitochondria/caspase-3-mediated cell death.

## Discussion

Infectious pancreatic necrosis virus (IPNV) is a fish pathogen E1-S of the IPNV Ab strain induces apoptotic cell death in CHSE-214 cells as suggested previously by Hong [30]–[33] and induces apoptotic death in zebrafish ZLE cells [34]. In this study, we examine the double-stranded RNA-IPNV and attempted to interpret how the virus triggers an PKR/PERK/eIF2α-mediated death signal for transcription and translation of a CHOP, a Bcl-2 suppressor leading to induction of host mitochondria-mediated cell death.

### RNA virus induces ER stress

Virus infection of mammalian and animal cells consists of a series of events, which involve in entry, RNA expression and processing, polypeptides synthesis and modification, genome replication, and maturation. On the other hand, as intracellular parasites, viruses rely on the utilization of cellular machinery and resource to complete their replication cycle, which stage viruses synthesize double-stranded RNA intermediates including RNA and DNA viruses and produce viral proteins within its host cells. Consequently, viral replication how to elicit cellular responses such as endoplasmic reticulum (ER) stress and interferon responses is still overcome. Therefore, it is not surprising that viruses have evolved various mechanisms to cope with these responses that limit or inhibit viral replication [45]. Recently, some viruses can induce ER stress as the tula virus [3], mouse retrovirus [13], hepatitis C virus [15], Japanese encephalitis virus [17] and betanodavirus [46] induce the ER stress response via upregulation of the ER chaperone protein GRP78. However, a few is known about ER stress responses to aquatic virus infection. In our system, we have found that IPNV infection could induce ER stress response via PKR/PERK/eIF2 signal death pathway for controlling the downstream CHOP and Bcl-2 expression.

### Dual kinase (PKR/PERK) modulate eIF2 phosphorylation

Unfolded proteins stimulate ER stress pathways, whereas dsRNA produced by viruses triggers the interferon pathway. These stress-responsive pathways converge at the subunit α of translation initiation factor 2 (eIF2α), which is essential for protein synthesis. Up to date, four different eIF2α kinases have been identified [47]–[48]. Amongst these kinases, PKR as well as PERK are activated by virus infection. Notably, PKR is a cytosolic and nuclear protein, which acts as an intracellular receptor for dsRNA produced by viral replication. In contrast, PERK is an ER-resident membrane protein that transmits ER stress signal. So PKR and PERK is activated by the virus in the cell is still not well defined. Several lines of evidence have indicated a link of viral replication to the PERK pathway [5], [14], [16], [49], [50], [51]. In the early phase of ER stress, accumulation of unfolded or misfolded protein activates PERK, which then phosphorylates eIF2α at serine 51. This leads to inhibition of general protein synthesis and reduces the protein load in ER. Furthermore, eIF2α phosphorylation also induces the expression of activating transcription factor 4 (ATF4), a transcription factor that stimulates the expression of C/EBP homologous protein (CHOP). In contrast, recent studies have shown that cytomegalovirus and African swine fever virus also perturb the PERK pathway [14], [51], [52]. Cytomegalovirus is a β-herpesvirus, which gene expression occurs in an ordered temporal pattern. Compared to the prototype herpes simplex virus-1, it is a slowly replicating virus. In cells infected with cytomegalovirus, PERK is not phosphorylated in the early phase, but as viral replication proceeds, in the level of PERK phosphorylation is increased later in infection [51]–[52]. In our system, we found that PERK autophosphorylation for phosphorylation of its substrate eIF2α mainly at middle stage (Fig. 2A, lane 3; 12 h p.i.), but phosphorylates the eIF2 at early replication stage (Fig. 2A, lane 2 and 4C:a, lane 3; at 6 h p.i.), which PKR and PERK is activated by the action of the virus in the cell to regulate the eIF2α phosphorylation at early and middle replication stages.

### CHOP induction affect Bcl-2 family protein expression

Furthermore, to upregulate CHOP protein expression via the PERK-eIF2α-ATF4 branch of the UPR signal is essential that promotes apoptosis [8], [45] and its suppressing by the activity of antiapoptotic Bcl-2 family proteins [21]. The C/EBP homologous protein (CHOP), also known as growth arrest and DNA damage-inducible protein (GADD153), is a dominant-negative inhibitor of the CCAAT/enhancer-binding proteins. When expressed in mammalian cells, CHOP/GADD153 facilitates apoptosis [10]. The downstream targets of CHOP remain unknown, but CHOP-mediated apoptosis has been coupled to a pathway that suppresses Bcl-2 expression, depletion of intracellular glutathionine, and an increase of free radicals [10], [53], but this signal few case correlated to regulate mitochondria functions. In addition to CHOP, Japanese encephalitis virus infection also activates p38 MAPK [17]. Inhibition of p38 MAPK activity alleviates apoptosis induced by Japanese encephalitis virus. In our system, we found that IPNV-triggered ER stress signaling can activate eIF2α through PKR/PERK pathways and upregulation of CHOP, which also correlate to suppresses Bcl-2 family proteins expression at middle stages (12 h p.i.) (Fig. 3A, lane 4). On the other hand, these are very interesting that Bcl-2 family proteins Bcl-2, Mcl-1 and Bcl-xL are also upregulated by IPNV between early (6 h p.i.) to early-middle replication stage (9 h p.i.), which responses may prime by host, but at 12 h p.i. (middle stage) are dramatically decreased their proteins expression (Fig. 3A, lanes 2–3). Furthermore, CHOP-mediated suppresses the Bcl-2 family proteins expression is modulated by GRP78 inhibitor VT (Fig. 5B, lane 3; [54]), which reduces ER stress response and corresponded to enhanced cell survival (Fig. 5F–G). On the other hand, Birnavirus, infectious disease virus (IBDV) that the signaling pathways are known. IBDV causes apoptosis and it employs p38 MAPK signal transduction machinery to elicit macrophage and IBDV-induced apoptosis is caspase-dependent via caspase-3 and -9 activations [55]–[56].

The mitochondrion functions as a central integrator of pro-death stimuli [22] by sequestering apoptogenic proteins such as cytochrome *c*, Smac/DIABLO, apoptosis inducing factor, and endonuclease G in the intermembrane space, and releasing these factors into the cytosol on exposure to proapoptotic signals [57]–[58]. The mitochondrial membrane potential (MMP) loss leads to activate the caspase-9 of the downstream activator of apoptosis [57]–[58]. MMP loss can affect both the inner and outer mitochondrial membranes, and precedes the signs of necrotic or apoptotic cell death, including the apoptosis-specific activation of caspases [22]. Hence, the mitochondrion functions as a central integrator of pro-death stimuli, joining together various types of proapoptotic signals into a common caspase-dependent pathway [22]. In our system, we found that IPNV-induced ER stress signaling can regulate the mitochondria function. This death signal may suppresses Bcl-2 family proteins expression via CHOP upregulation that also leads to loss the MMP (Fig. 6A) and produces cytochrome *c* release (Fig. 6C), caspase-3 (Fig. 6D) activation, which also blocked by GRP78 inhibitor (Fig. 6) that received a consistent results.

**Figure 7:**
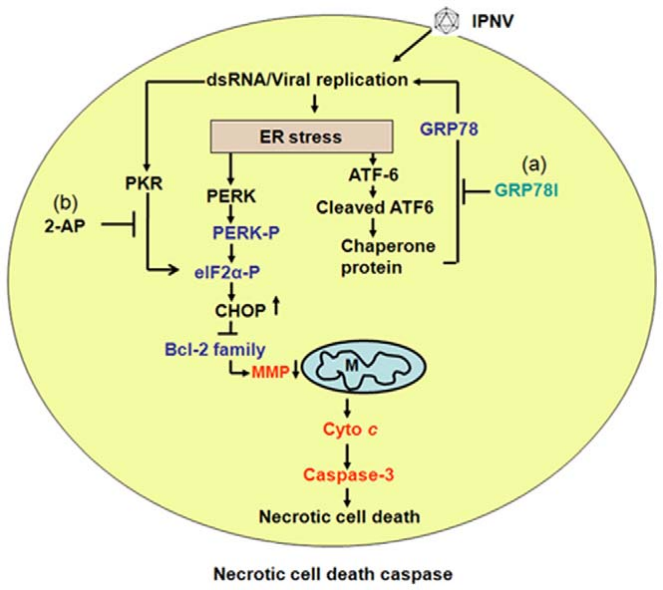
IPNV induces ER stress-mediated host cell death cascade. IPNV infection and early replication causes an ER stress response upon entry and the primary replication stage. Then, viral replication triggers an ER stress induction stage that includes: 1) activating ATF6 sensor that up-regulate chaperone protein GRP78; 2) PKR can phosphorylate the eIF2α; 3) PERK sensor is autophosphorylated, which can enhance eIF2α phosphorylation. Further, the PKR/PERK ER stress signal can induce CHOP up-regulation, which may correlate to Bcl-2 family members downregulation such as Bcl-2, Mcl-1 and Bcl-xL at early (0–6 h pi) and middle (6–12 h pi) replication stages. Finally, IPNV infection induce PKR/PERK-mediated downredulate Bcl-2 expression and MMP loss, which combined the death signals for triggering necrotic cell death at the mitochondrial dysfunction stage at middle and late (12–24 h pi) replication stages. Cell death could be modulated by: (a) VT to inhibition of GRP78 expression and (b) 2-AP to reduce PKR activation.

We schematically depict the steps to summarize the process of IPNV-induced ER stress signaling for induction of mitochondria mediated cell death. As shown in Fig. 7, IPNV entering and replication in cytoplasm and induces the ER stress response for upregulation of GRP78. Then, eIF2α should be phosphorylated by PKR through dsRNA manner at early-middle replication stage (6 h p.i.) that eIF2α phosphorylation modulated by GRP78 inhibitor VT and PKR inhibitor 2-AP. Then, this ER stress signaling can induce of CHOP upredulation and reduce the Bcl-2 family downregulation such as Bcl-2, Mcl-1 and Bcl-xL. Moreover, downregulation of Bcl-2 family by eIF2α/CHOP signals is correlated to disrupt mitochondria function for cyto *c* releasing and caspase-3 activation. Our finding may provide new insights into RNA virus activate ER stress and host interaction on understanding molecular disease mechanism.
